# Stroke Exacerbates Respiratory Disorder and Cognition Impairment in Mice with Cerebral Amyloid Angiopathy

**DOI:** 10.14336/AD.2025.0474

**Published:** 2026-05-25

**Authors:** YuXing Zhang, Ahmad El Hamamy, Zahid Iqbal, Arya Ranjan, Destiny Sumani, Hung Wen Lin, Louise D. McCullough, Jun Li

**Affiliations:** Department of Neurology, McGovern Medical School, University of Texas Health Science Center at Houston, TX,77030, USA

**Keywords:** cerebral amyloid angiopathy, vascular dementia, breathing dysfunction, cognition impairment, retrotrapezoid nucleus

## Abstract

Stroke is a known risk factor for dementia. Most Alzheimer’s patients exhibit mixed neuropathology, with evidence of both ischemic damage and amyloid-beta (Aβ) plaque accumulation. Breathing disorders, such as apnea, are also associated with cognitive dysfunction and dementia progression. We hypothesized that stroke exacerbates respiratory dysfunction and cognitive impairment in Tg-SwDI mice, a model of cerebral amyloid angiopathy (CAA). Female CAA mice (11–13 months old) underwent permanent distal middle cerebral artery occlusion (pd-MCAO) surgery, with age- and sex-matched wild-type and sham-operated controls. Cognitive assessments included the Barnes maze, and novel object recognition test (NORT). Respiratory metrics were quantified using whole-body plethysmography, while immunohistochemistry measured Aβ deposition in the hippocampus and cortex, astrocytic markers (C3⁺GFAP⁺ for A1; S100A10⁺GFAP⁺ for A2) in the retrotrapezoid nucleus (RTN), and lymphatic vessel area (LYVE1) in deep cervical lymph nodes (dCLNs). Aβ in cerebrospinal fluid was also assessed. CAA mice without stroke exhibited higher apnea rates and impaired cognitive performance compared to wild-type controls. Stroke further increased apnea events and worsened Barnes maze escape latencies in CAA mice. Molecular analysis revealed an increase in GFAP as well as in A1 astrocytes and a reduction in A2 astrocytes in the RTN following stroke. Additionally, stroke accelerated Aβ deposition in the hippocampus and cortex while reducing Aβ clearance via cerebrospinal fluid and dCLNs. These findings suggest that stroke exacerbates respiratory dysfunction, impairs glymphatic-lymphatic clearance, and accelerates cognitive decline in CAA mice. Targeting post-stroke respiratory dysfunction may offer therapeutic potential for mitigating ischemic damage in dementia patients.

## INTRODUCTION

The expanding aging population has led to a rise in neurological disorders, especially amyloid-related conditions such as cerebral amyloid angiopathy (CAA) and ischemic stroke, posing a significant public health challenge [1-3]. CAA is characterized by the deposition of amyloid-beta (Aβ) around cerebral blood vessels and contributes to an increased risk of stroke during its progression. CAA-related vascular dysfunction can further impair post-stroke recovery by disrupting cerebral blood flow and neurological function [4]. On the other hand, stroke may exacerbate Aβ accumulation, further deteriorating cognitive function [5]. Specifically, CAA is a degenerative vasculopathy that involves the progressive deposition of Aβ protein within the walls of leptomeningeal and cortical blood vessels, particularly in regions such as the cortex and hippocampus, which play essential roles in cognition, memory, and learning [6]. This accumulation leads to vascular fragility, impaired cerebrovascular autoregulation, and chronic hypoperfusion, contributing to the development of cerebral infarctions, microbleeds, and vascular-related cognitive decline [7, 8]. Over time, accumulation of Aβ exacerbates neurovascular dysfunction, promotes neuroinflammation, and disrupts the blood-brain barrier, further amplifying its pathological impact on brain [9]. Although previous studies have primarily examined the hemorrhagic effects of CAA, recent research emphasizes the need to explore its ischemic manifestations [4, 10, 11].

CAA is an important contributing factor in stroke occurrence. Clinical evidence indicated that CAA was detected in 291 of 1,620 patients with ischemic stroke/TIA [12]. The underlying vascular pathology CAA increases the risk of cerebral infarctions and worsens post-stroke outcomes [12]. Individuals with CAA are more susceptible to ischemic strokes due to chronic hypoperfusion and impaired vascular reactivity [12, 13]. Furthermore, CAA-related small-vessel disease leads to silent infarcts and white matter degeneration, both of which contribute to progressive cognitive decline [14]. The presence of CAA also complicates ischemic stroke recovery by limiting vascular repair, exacerbating neuroinflammation, and increasing the likelihood of subsequent cerebrovascular events. CAA has been increasingly linked to respiratory dysfunctions, especially sleep apnea, which involves repeated interruptions in breathing during sleep. This condition not only raises cardiovascular risks but also has significant neurological effects, adding to the complexity of managing these disorders [15-17].

Our previous research demonstrated that CAA mice showed increased apnea episodes, and reduced respiratory rate, which, at least in part, are due to enhanced TGF beta signaling and gliosis in retrotrapezoid nucleus (RTN) in the brainstem [11]. The RTN is a structure in the ventral medulla of the brainstem that contains specialized glutamatergic neurons and plays a key role in CO₂ sensing and regulation of respiratory rhythm [18, 19]. It regulates the rodent respiratory system through negative feedback mechanism aimed at maintaining blood gas homeostasis by facilitating O₂ uptake and CO₂ elimination [20]. Our previous work showed the RTN gliosis plays a causal role in CAA breathing disorder and cognitive impairment progression. However, how stroke impacts RTN pathology and breathing disorder in CAA is unknown. Impaired respiratory function has been closely associated with cognitive impairment and abnormal clearance of Aβ, particularly through dysfunction of the glymphatic system, a brainwide fluid transport pathway responsible for clearing metabolic waste [21]. After stroke, the glymphatic system exhibits marked dysfunction, characterized by reduced interstitial fluid clearance and impaired cerebrospinal fluid transport, which contributes to the accumulation of neurotoxic proteins such as Aβ [22]. These findings underscore the importance of maintaining proper respiratory regulation, especially in brainstem structures like the RTN, in supporting efficient glymphatic function and mitigating the progression of CAA.

In this study, we hypothesized that the stroke in mice with CAA will further disrupt respiratory function, increase brain stem RTN astrogliosis, impair glymphatic clearance, and promote Aβ accumulation in the brain, leading to exacerbated cognitive impairment.

## METHODS AND MATERIALS

### Animals

Female adult (11–13 months old) Tg-SwDI (CAA model) mice and age- and sex-matched C57BL/6 WT mice were bred in-house from mice obtained from Jackson Laboratories and used in all experiments. Mice were kept in a climate-controlled vivarium, 5 per cage, on a 12-hour light/dark cycle with unrestricted access to food and water. Each mouse in a cage was randomly assigned a number from 1 to 5 by a lab member not involved in the experiment procedure. A random number generator was then used to assign each number to either the control or treatment groups.

### Animal ethics declaration

All experiments were performed according to NIH guidelines for the care and use of animals in research and under protocols approved by the University of Texas Health Science Center Houston Institutional Animal Care and Use Committee.

### Permanent distal middle cerebral artery occlusion (pd-MCAO) model

Permanent focal cerebral ischemia was induced by permanent occlusion of the right distal middle cerebral artery (MCA) using an electro-cauterizer [23]. The distal MCA was accessed via a craniotomy and permanently occluded just proximal to the anterior and posterior branches. Mice were anesthetized with isoflurane (4% induction and 2% maintenance in airflow) during the surgery. The body temperature (rectal probe) was maintained at 37 °C during surgery. Bupivacaine (1 ml/kg of 0.25% solution, s.c.) was injected prior to skin incision for pain management. A skin incision was made between the ear and eye, and the temporal muscle was detached from the skull to locate the MCA beneath the transparent skull. A small craniotomy was then generated over the MCA using a micro-drill to provide access to the artery. An Accu-temp variable high temperature cautery was used to permanently occlude the MCA. After surgery, mice received heat support for 2 h. Sham controls underwent the same procedure, except for the cauterization of the MCA.

### Whole-body Plethysmography

Breathing patterns were evaluated using a whole-body plethysmography system specifically designed for mice, following our previously described protocol [24]. Mice were first placed in the chamber and given 30 minutes to acclimate. Respiratory measurements, including tidal volume, respiratory rate, and minute ventilation, were then recorded over a 20-minute period after a total of 1 hour of habituation. To detect apneic events, we used a dual-threshold method. An apnea was defined as a breathing pause lasting at least twice as long as the average baseline breath duration, in line with established criteria [24]. Additionally, to qualify as apnea, the breathing pause had to longer than 0.5 seconds, based on prior studies [25]. For each mouse, apnea frequency was calculated as the number of apneas per minute.

### Barnes Maze

Mice underwent Barnes Maze to assess hippocampal-dependent spatial memory, as previously described [26, 27]. The maze consisted of a circular flat platform with 20 evenly spaced holes (diameter-5cm) around the perimeter, one of which has a dark secure escape chamber underneath as the escape hole, allowing mice to escape the light and noise stimulations. Mice were trained for 4 consecutive days (training days) to locate the escape box positioned underneath one of the holes. If the mouse failed to find the dark box within 5 minutes, it was gently guided toward the box. The maze was cleaned thoroughly after each mouse with 70% isopropyl alcohol to eliminate odor cues. Latency to find the escape hole and the number of errors were recorded for each trial. On the fifth day (testing day), a test trial was performed to evaluate memory retention.

### Novel Object RecognitionTest (NORT)

For testing cognitive memory function, mice were tested using Novel Object Recognition Test (NORT), as previously described [28]. Briefly, mice were put in an open field arena for a 5-min habituation period on day 1. On day 2, mice were placed in the same arenas with two identical objects and left to explore both objects. On the third day, one of the familiar objects was switched with a novel object. Testing stopped in all trials after each mouse reached a threshold of 30 seconds total exploration time between the two objects, and all trials were recorded and analyzed by EthoVision. The percentage of time spent with the novel object was calculated.

Percent of Time with Novel Object= (Time Spent with Novel Object \ Total Time Spent with Both Objects) ×100.

### CSF collection

Using previously published protocols, mice were deeply anesthetized by avertin and tightly secured in a stereotaxic frame to ensure the head was at a 120° angle to the neck [29]. The skin of the dorsal neck was shaved and cleaned with iodine and 70% ethanol. Using scissor and curved forceps, the muscle over the base of the skull was carefully removed, exposing the dura mater over the cisterna magna (which is triangular with 1-2 large basilar arteries in this area). Using the tip of a glass capillary, the membrane was punctured until resistance was encountered, allowing CSF to be automatically drawn into the tube. The CSF samples were then centrifuged at 2000g for 10 minutes to eliminate any blood materials. After centrifugation, the CSF was promptly stored at -80℃ for further analysis.

### Quantification of Aβ peptides Enzyme-linked immunosorbent assay

To determine the amount of mouse Aβ42 peptides, the major component of amyloid plaques, in mouse cerebral spinal fluid, commercial ELISA kits (Thermo Fisher Scientific, Waltham, MA, USA: KHB3442) were used according to the manufacturer’s instructions. Briefly, 6μL CSF was diluted in 54μL sample dilution. Then, according to the manufacturer’s instruction, the absorbance at 450nm was measured using a Bio-Rad 680 microplate reader (Biotek, USA).

### Immunostaining

Mice were deeply anesthetized by avertin (300 mg/kg) and then perfused transcardially with ice-cold phosphate-buffered saline (PBS), followed by 4% paraformaldehyde (PFA), in accordance with previous methods [30]. The hemispheres and brainstems were extracted, post-fixed in PFA for 24 hours, and cryoprotected in a 30% sucrose solution containing 0.02% sodium azide for 48 hours. Hemisphere and brainstems were sectioned into 30-micron slices using a sliding microtome. The sections were rinsed with PBS and permeabilized for 15 minutes in PBS with 0.3% Triton X-100. Next, they were blocked for 1 hour in a solution containing 5% donkey serum and 1% bovine serum albumin (BSA) in 0.3% Triton X-100. dCLVs slices were dewaxed, rehydrated, antigen retrieval and blocked as required. Sections were then incubated overnight at 4°C with primary antibodies against Aβ (1:300, Abcam, Cat# ab201060), Phox2B (1:20, Bio-Techne, Cat# AF4940), GFAP (1:200, Novus, Cat# NB100-53809), C3 (1:200, Abcam, Cat# ab97462) S100A10 (1:200, Invitrogen, Cat# PA5-95505), LYVE1(1:200, CST, Cat# E3L3V) and in the blocking solution. After primary incubation, sections were washed with PBS and incubated with appropriate secondary antibodies (1:500, Abcam, anti-Goat Cat# ab150131, anti-Rabbit Cat# ab150073, anti-Mouse Cat# ab150115) for 1 hour at room temperature in the dark. Sections were finally mounted on slides, counterstained with DAPI, and coverslipped. For negative controls, sections were processed without primary antibodies to verify specificity and eliminate non-specific binding of secondary antibodies. Imaging was conducted using the Leica Thunder Imaging System, and quantification was performed with Fiji software version 2.15.1.

### Statistical analysis

All statistical analyses were performed using SPSS (Statistical Package for the Social Sciences, version 27.0). Data are expressed as the mean ± S.E.M., and *P* < 0.05 was considered statistically significant. All data were tested for normality using the Shapiro-Wilk test. Post hoc comparisons following a significant Kruskal–Wallis test were performed using Mann–Whitney U tests (non-normal distribution). For comparisons involving three or more groups, one-way analysis of variance (ANOVA) followed by the LSD post hoc test were employed to analyze the data pertaining to multiple groups. All statistical charts were drawn using GraphPad Prism 10.0 and Photoshop 2021.

**Figure 1.**
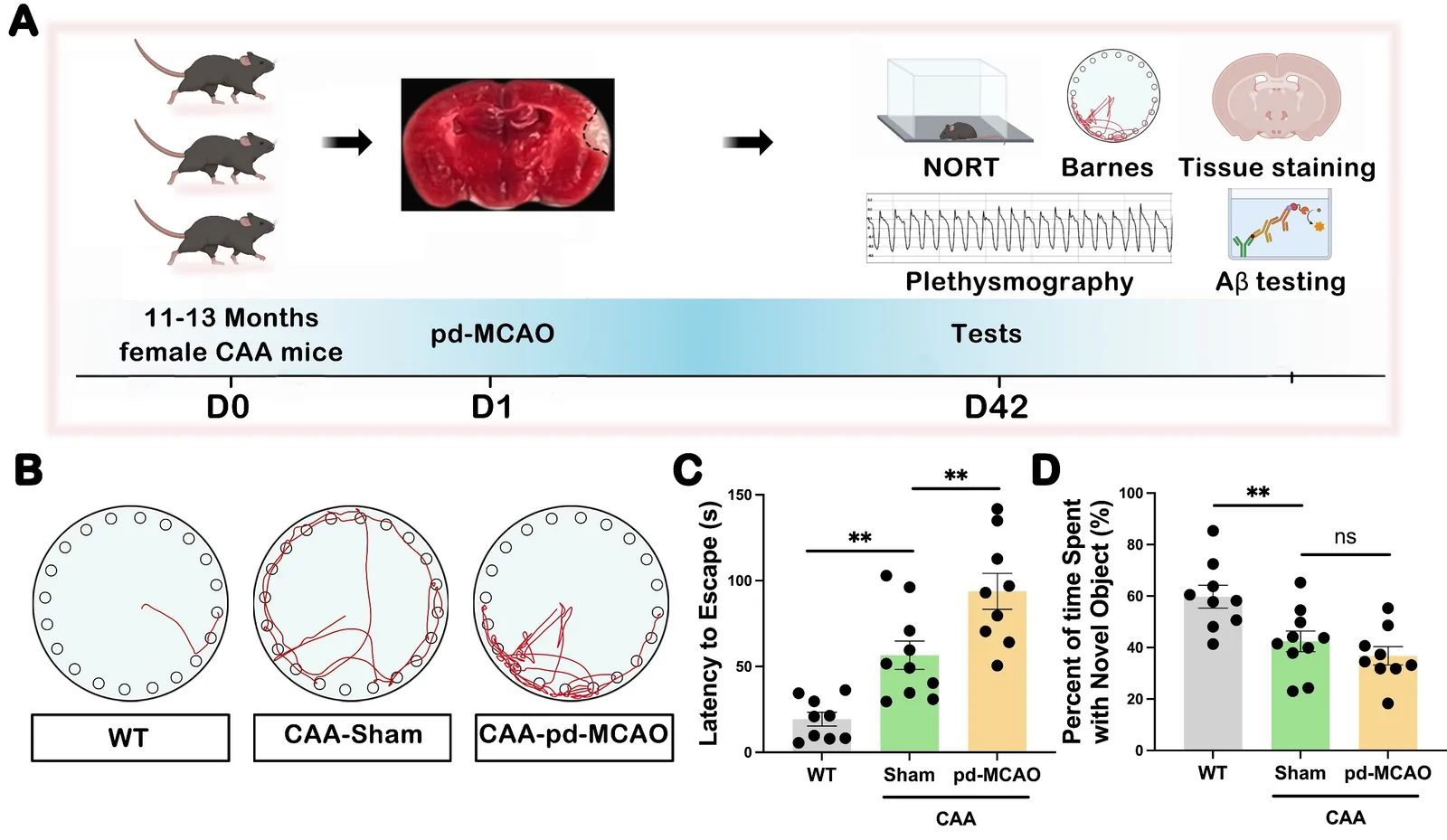
**Distal stroke exacerbates cognitive impairment in CAA mice on day 42 post-stroke. (A)** Schematic description of the experimental timeline for the pd-MCAO model, behavioral tests, whole-body plethysmography test, and biological assessments on days 42 post-stroke. **(B)**Representative trajectories and quantification of **(C)** latency to escape time in Barnes Maze test (F (2,25) =20.563, *P*＜0.001, n=9 for WT and CAA-pd-MCAO group, n=10 for CAA-Sham group). **(D)**Quantification of percent of time spent with novel object (F (2,25) =8.63, *P*=0.001, n=9 for WT and CAA-pd-MCAO group, n=10 for CAA-Sham group). * *P* <0.05; ** *P* <0.01; *** *P* <0.001. ns: non-significance. The data are shown as the mean ± SEM. After performing the Shapiro-Wilk normality test to examine the normal distribution, One-way analysis of variance (ANOVA) was employed to analyze the data pertaining to multiple groups, subsequently, multiple comparisons were conducted using the uncorrected Fisher's LSD test.

## RESULTS

### Distal Stroke Exacerbates Cognition Impairment in Mice with Dementia

To determine whether distal MCAO affected cognitive function in CAA mice, 11-13-months old WT and CAA female mice were subjected to permanent distal-MCAO or Sham surgery. On days 42 post-surgery, Barnes maze, Novel objects recognize test (NORT) were performed in each group to identify the cognitive function (Fig. 1A).

As we expected, CAA mice (Tg-SwDI) with sham surgery (a small craniotomy using a micro-drill without cauterization of the MCA) showed worse cognition, with a prolonged latency to find out the escape chamber compared to WT mice (56.5±8.2 vs. 19.3±4.0, *p*=0.002, n=10 and 9; Fig. 1C) in the Barnes Maze. In addition, representative search trajectories revealed that CAA-Sham mice spent more time exploring the maze, displaying more disorganized and prolonged searching behavior compared to WT mice (Fig. 1B). CAA mice with stroke took a longer time to find the escape chamber than the CAA sham group reflecting stroke-induced memory impairment (93.7±10.4 vs. 56.5±8.2, *p*=0.003, n=9 and 10; Fig. 1C). Compared to CAA-Sham mice, CAA with stroke mice exhibited markedly more disorganized and inefficient search patterns during the Barnes maze test, characterized by increased random movements, frequent re-crossing of the center area, and reduced peripheral hole exploration, indicating severe impairment in spatial memory and goal-directed navigation (Fig. 1B). In the NORT, the percentage of time spent with novel object in CAA mice was decreased compared to WT mice (42.4±4.0% vs. 59.7±4.4%, *p*=0.004, n=10 and 9; Fig. 1D), while there was a non-significant reduced novel object interaction time between CAA mice and CAA mice with pd-MCAO surgery (36.8%±3.54 vs. 42.4±4.0, *p*=0.33, n=9 and 10; Fig. 1D).

**Figure 2.**
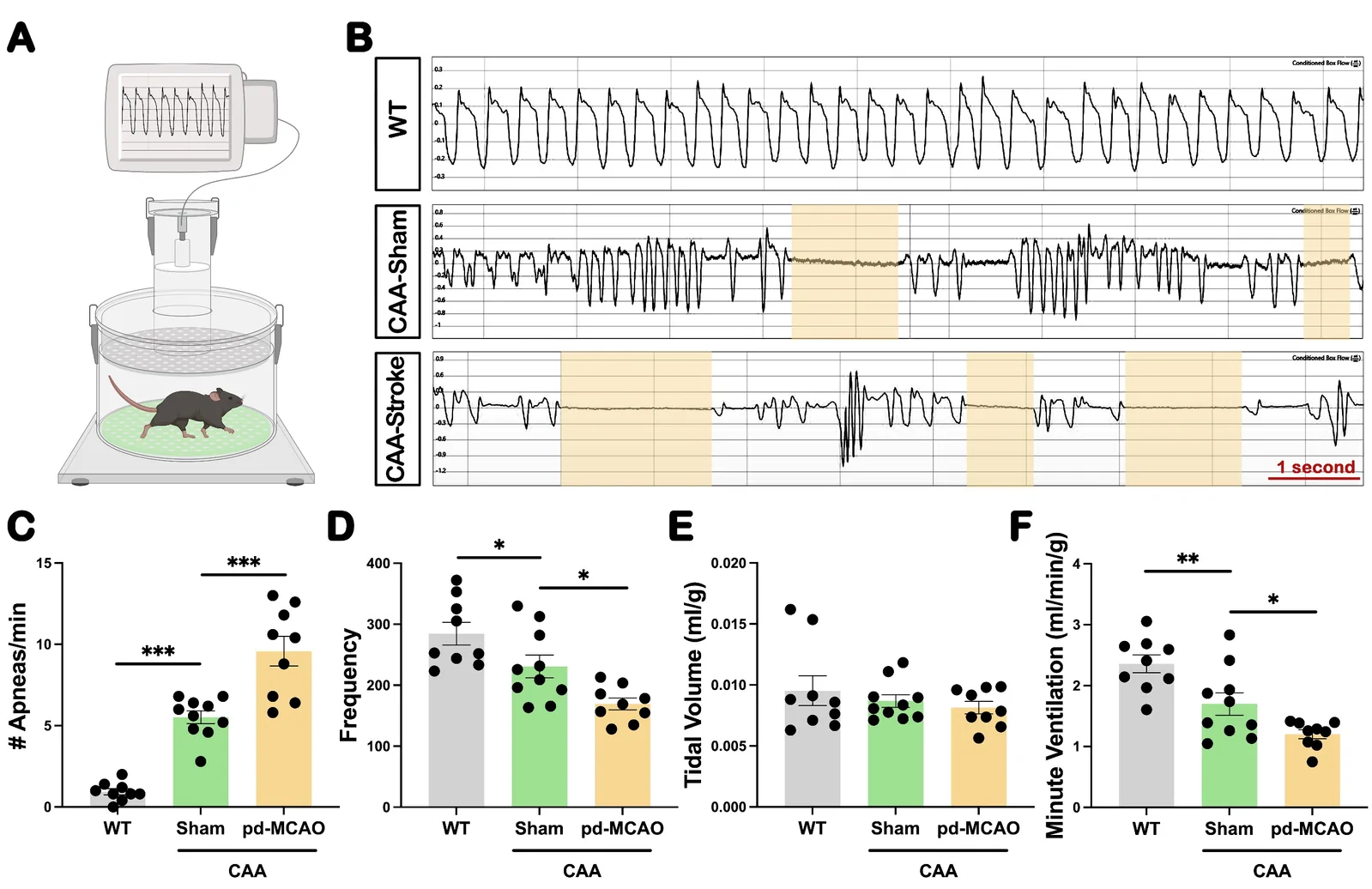
**Distal Stroke Exacerbates Respiratory Disorder in CAA mice on day 42 post-stroke. (A)** Schematic description of the Whole-body plethysmography test for the pd-MCAO model. **(B)**Representative respiratory waveform tracings of mice, **(C)** number of apneas per minute (F (2,18) =33.56, *P*＜0.001), **(D)**respiratory frequency per minute (F (2,18) =12.82, *P*＜0.001), **(E)**total tidal volume(TV; mL/L, F (2,18) =0.6638, *P*=0.5270) and **(F)** minute ventilation per minute/gram (MV; mL/L, per min, per g), F (2,18) =16.85, *P*＜0.001 of WT, CAA, CAA-Stroke group in whole body plethysmography (n= 9 for WT and CAA-pd-MCAO group, n=10 for CAA-Sham group).Scale bar = 1 second. * *P* <0.05; ** *P* <0.01; *** *P* <0.001. ns: non-significance. The data are shown as the mean ± SEM. After performing the Shapiro-Wilk normality test to examine the normal distribution, One-way analysis of variance (ANOVA) was employed to analyze the data pertaining to multiple groups, subsequently, multiple comparisons were conducted using the uncorrected Fisher's LSD test.

### Distal Stroke Exacerbates Respiratory Dysfunction in Mice with Dementia

Disordered breathing occurs frequently in patients with amyloid related dementia [31], in turn, apnea increases the Aβ burden by impairing glymphatic clearance ability and is an independent risk factor for the development of cognitive dysfunction [32]. Previous studies in our lab showed that transient proximal MCAO produces stroke-induced respiratory dysfunction (SIRD) characterized by hypoventilation and apneas in young mice [24]. To determine whether distal MCAO affects respiratory function in older CAA mice, we performed whole body plethysmography to evaluate respiratory function. On day 42 post-stroke, mice underwent plethysmography to evaluate the SIRD phenotype (Fig. 2A). Representative whole-body plethysmography recordings indicated that WT mice exhibited regular, rhythmic breathing cycles. In contrast, CAA-Sham mice showed intermittent breathing irregularities with occasional pauses. CAA-Stroke mice demonstrated pronounced respiratory instability, characterized by frequent and prolonged apneic episodes, highlighted by the yellow shaded areas, indicating impairment in respiratory rhythm maintenance (Fig. 2B). Compared to WT mice, CAA mice exhibited an increased apneas (5.8±0.26 vs. 1.04±0.11, *p*=0.0003, n=7 and 5; Fig. 2C), with suppressed respiratory frequency (197.7±10.1 vs. 241.0±5.7, *p*=0.009, n=7 and 5; Fig. 2D). Tidal volume (TV; mL/g) reflects the capacity for gas exchange in a single breath, and minute ventilation (MV; mL/min per g) reflect the overall ventilation efficiency and metabolic demand. CAA mice showed no changes in TV (0.0091±0.0006 vs. 0.0095±0.0017, *p*=0.078, n=7 and 5; Fig. 2E) but had a significant decrease in MV (1.4±0.1 vs. 2.1±0.2, *p*=0.004, n=7 and 5; Fig. 2F). CAA mice with stroke had a significant increase in the number of apneic events compared to sham CAA mice under room air conditions (9.5±0.91 vs. 5.8±0.26, *p*=0.0009, n=9 and 7; Fig. 2C), and stroke further decreased respiratory frequency in CAA mice (169.3±9.6 vs. 197.7±10.1, *p*=0.04, n=9 and 7; Fig. 2D) or MV (1.2±0.1 vs.1.4±0.1, *p*=0.2, n=9 and 7; Fig. 2F) in CCA mice, without changes in TV (0.0081±0.0005 vs. 0.0091±0.0006, *p*=0.41, n=9 and 7; Fig. 2E). These results showed that disordered breathing is a common occurrence in mice with amyloid-β related CAA, and stroke further exacerbates these respiratory impairments.

**Figure 3.**
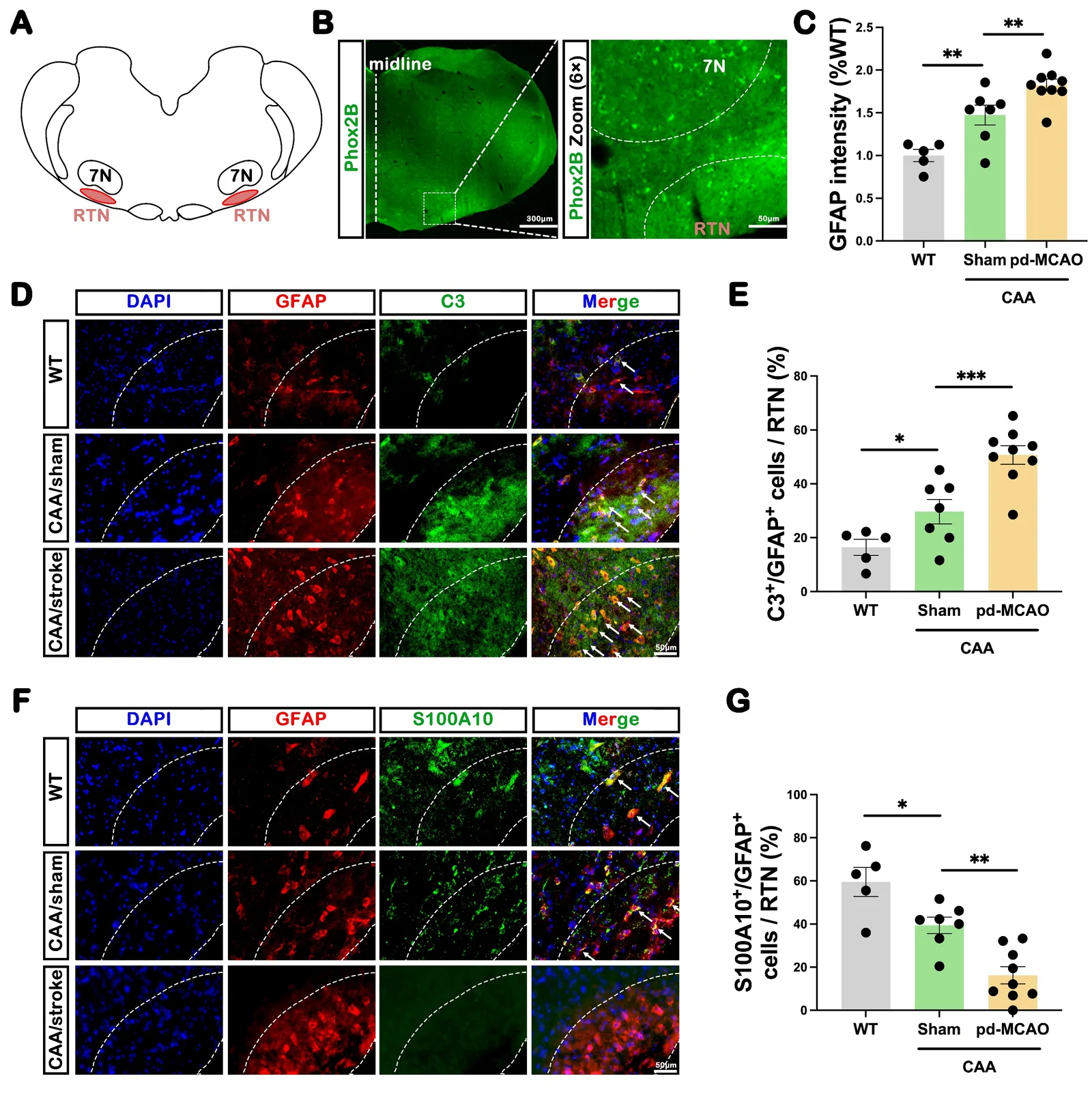
**Stroke increases astrocytic gliosis and induced astrocytic polarization in RTN. (A)** RTN chemo-sensitive phox2B-positive neurons (area circumscribed by red solid line) are located ventromedial to the VII, medial to the pyramidal tract. **(B)**Histological representation of the RTN location within the brainstem, illustrating the specific area analyzed in both WT, CAA, and stroke mice, in relation to the 7th Facial Nucleus (7 N), Scale bar = 300μm/50 μm (zoom). **(C-G)**Images show the GFAP expression in each group(F (2,18) =19.043, *P*＜0.001), and dual-IF staining for GFAP with C3 (F (2,18) =19.851, *P*＜0.001 ), GFAP with S100A10 (F (2,18) =21.306, *P*＜0.001) in RTN from WT, CAA, and stroke mice at day 42 post-ischemia (n= 5 for WT group, n=7 for CAA-Sham group and n=9 for CAA-pd-MCAO group). Scale bar = 50 µm. * *P* <0.05; ** *P* <0.01; *** *P* <0.001. ns: non-significance. The data are shown as the mean ± SEM. After performing the Shapiro-Wilk normality test to examine the normal distribution, One-way analysis of variance (ANOVA) was employed to analyze the data pertaining to multiple groups, subsequently, multiple comparisons were conducted using the uncorrected Fisher's LSD test.

### A1/A2 subtype reactive astrocytes were induced in the RTN following distal ischemic stroke

To evaluate astrocyte reactivity and phenotypic transformation in the RTN region, we performed immunofluorescence staining for GFAP, C3 (a marker of A1 neurotoxic astrocytes), and S100A10 (a marker of A2 neuroprotective astrocytes). To precisely locate the RTN region for subsequent analyses, we first referred to anatomical landmarks. The RTN is situated ventral to the facial motor nucleus (7N) in the ventral brainstem (Fig. 3A). Immunofluorescence staining for Phox2B, a transcription factor selectively expressed in RTN neurons, was performed to identify the RTN region. As illustrated in Figure 3B, Phox2B-positive cells were predominantly localized in the ventral area beneath the 7N, confirming the accurate identification of the RTN for further quantification. GFAP expression, indicative of reactive astrogliosis, was significantly upregulated in CAA mice compared to WT controls and further elevated in the CAA/stroke group (Fig. 3C). As shown in Figure 3D and Figure 3E, CAA mice exhibited increased numbers of C3⁺/GFAP⁺ reactive astrocytes compared to WT in the RTN region (29.6%±4.48 vs. 16.4%±2.97, p=0.039, n=7 and 5), and this was further elevated following stroke (50.7%±3.44 vs. 29.6%±4.48, p＜0.001, n=9 and 7). Conversely, the proportion of S100A10⁺/GFAP⁺ astrocytes (Fig. 3F) was significantly reduced in the CAA group compared to WT (39.3%±3.79 vs. 59.5%±6.74, p=0.011, n=7 and 5), and further decreased after stroke (16.2%±3.98 vs. 39.3%±3.79, p=0.0013, n=9 and 7, Fig. 3G), suggesting a transformation toward a more neurotoxic astrocyte phenotype after stroke in CAA mice. These findings indicate that stroke aggravates astrocytic reactivity and promotes A1-like transformation in the RTN region. The imbalance between A1 and A2 astrocyte populations may contribute to respiratory and cognitive dysfunction after stroke, contributing to comorbidity in mice with amyloidosis.

### Distal Stroke increase amyloid β deposition in the cortex and hippocampus

**Figure 4.**
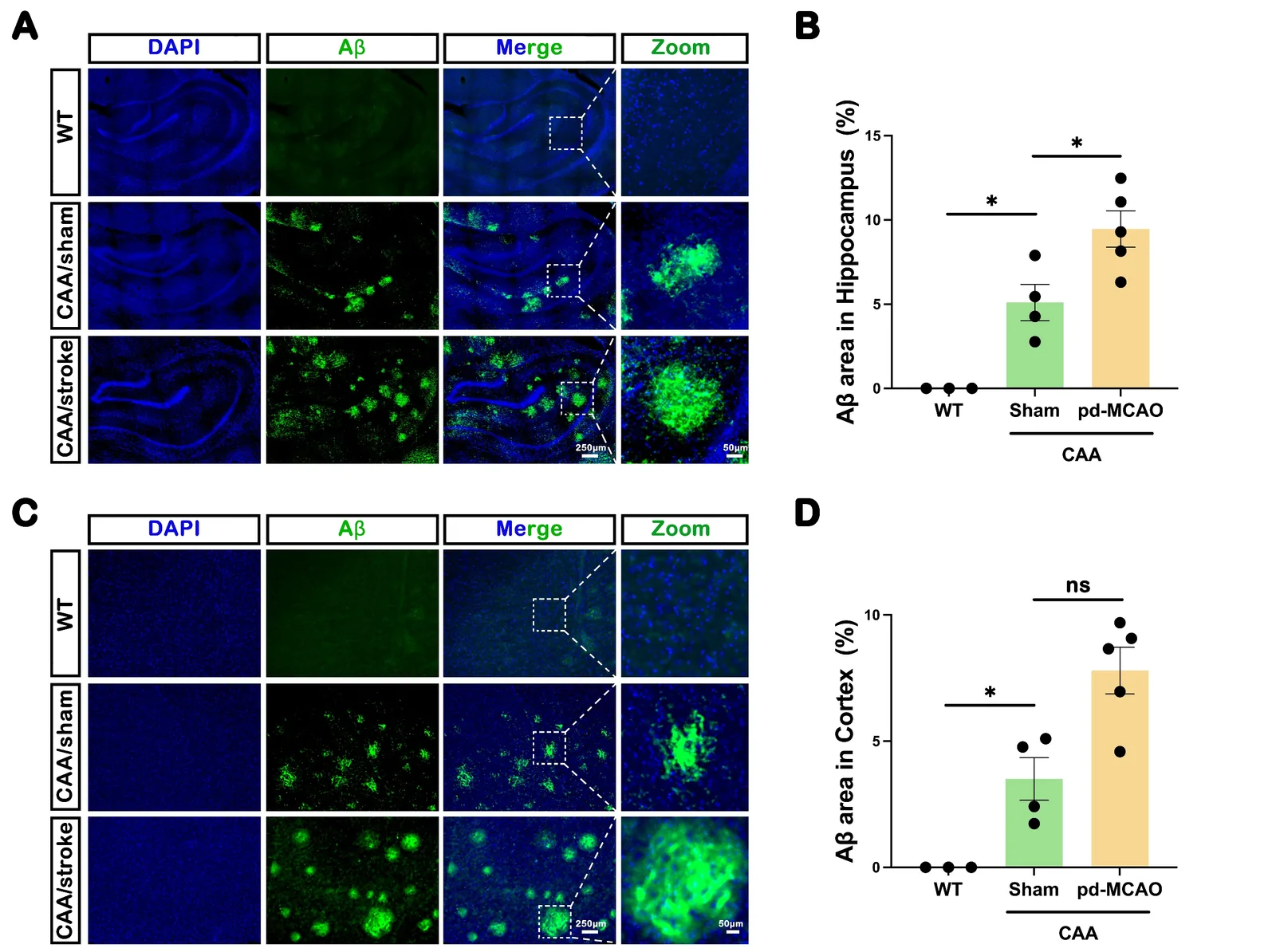
**Stroke increase the Aβ deposits in the cortex and hippocampus. (A)** Representative images of the Aβ in hemisphere and **(B)**percentage area ofAβ in the brain(H (2) = 9.16, *P*＜0.05), scale bar = 200μm/50μm(zoom).**(C)**Representative images of the Aβ in cortex and **(D)**percentage area ofAβ in the cortex (H (2) =8.566, *P*＜0.05), scale bar = 200μm/50μm(zoom), (n= 3 for WT group, n=4 for CAA-Sham group and n=5 for CAA-pd-MCAO group). * *P* <0.05; ** *P* <0.01. ns: non-significance. non-parametric Kruskal–Wallis H test was applied to determine overall differences among groups. Subsequently, pre-specified pairwise comparisons were conducted using the Mann–Whitney U test without correction for multiple comparisons. The significance level was set at *P* <0.05, and no correction was applied due to the limited number of comparisons and predefined hypotheses.

Our behavioral and plethysmography resultsshowed a significant change in cognitive impairment and breathing dysfunction in CAA mice which is exacerbated by stroke. Since apnea is an independent risk factor for vascular dementia [33], and in order to explore the underlying mechanisms of cognitive impairment as well as the changes in Aβ deposition following respiratory dysfunction, we performed immunofluorescence staining for Aβ in the cortex and hippocampus [34]. Aβ plaque burden was significantly increased in both the hippocampus (*p* < 0.05, Fig. 4A-B), and the cortex (*p* < 0.05, Fig. 4C-D) in the CAA group compared with the WT group, and stroke markedly upregulated the elevated Aβ plaque burden in the hippocampus (9.4%±1.08 vs. 5.0%±1.08, *p*=0.027, n=5 and 4) and no signiticant increase in the cortex Aβ plaque burden was observed (7.7%±0.92 vs. 3.5%±0.84, *p*=0.05044, n=5 and 4), as compared with the CAA sham group.

These findings suggest that respiratory dysfunction and stroke synergistically aggravate Aβ deposition in critical cognitive regions such as the cortex and hippocampus. Therefore, we investigated the mechanisms responsible for impaired Aβ clearance. Previous studies have highlighted the essential role of the meningeal lymphatic system and cervical lymph nodes in facilitating the drainage of interstitial and cerebrospinal solutes, including Aβ [35, 36]. Therefore, in the next phase of our study, we aimed to assess lymphatic flux function, particularly focusing on cervical lymph node activity and cerebrospinal fluid Aβ levels, to further elucidate the clearance pathway disruptions contributing to Aβ accumulation and cognitive decline.

### Stroke decreases glymphatic clearance in mice with dementia

**Figure 5.**
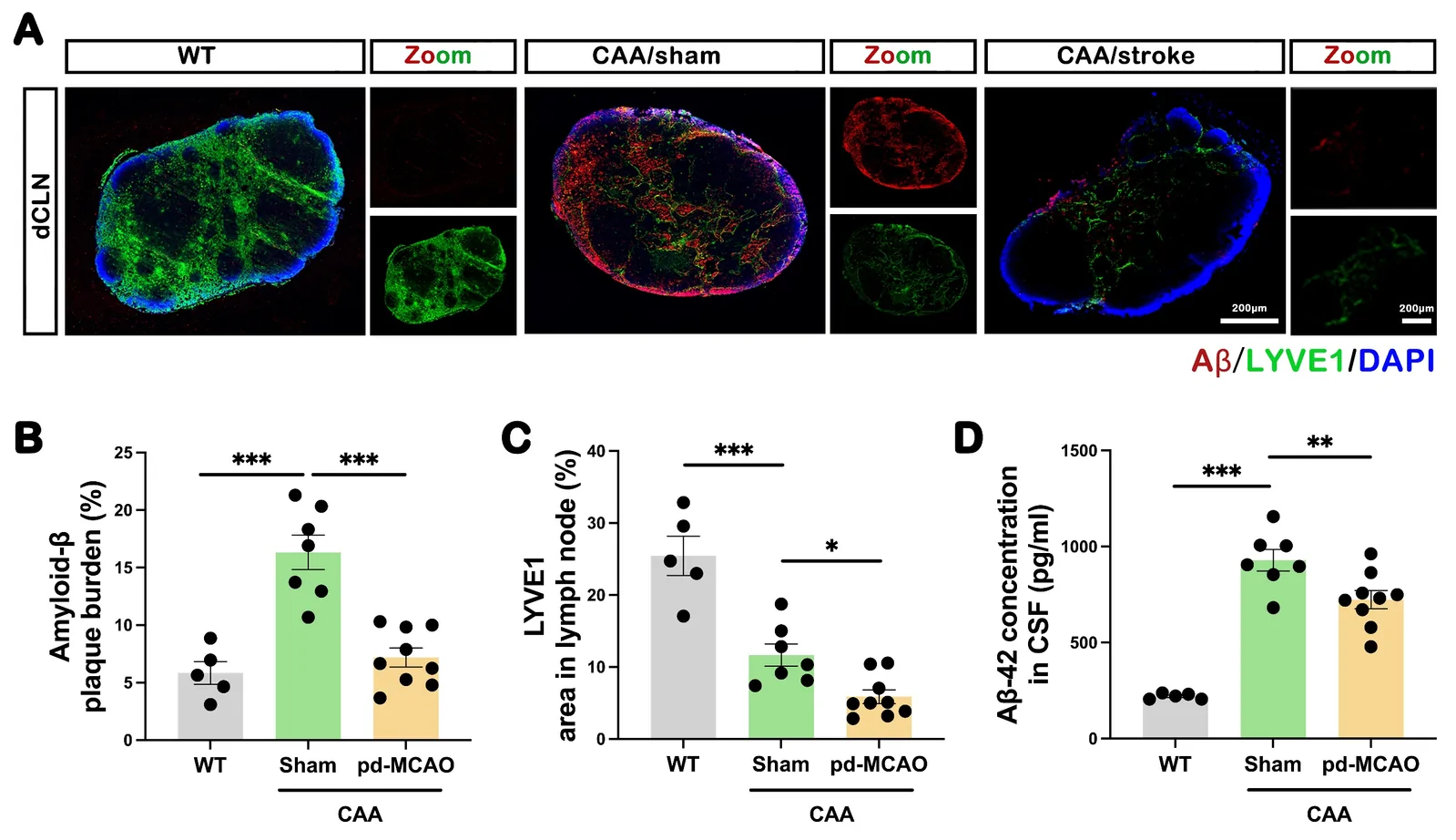
**Stroke decreased the drainage function of dCLVs in mice with CAA. (A)** Representative images of the Aβ (red), LYVE1 (green) and DAPI (blue) in the dCLVs, **(B)**quantification ofpositive area**of**Aβ plaque (%) (F (2,18) =24.21, *P*＜0.001) and **(C)** positive area of LYVE1 (%) in the dCLVs (F (2,18) =35.209, *P*＜0.001 ), (n= 5 for WT group, n=7 for CAA-Sham group and n=9 for CAA-pd-MCAO group). **(D)** Concentration of Aβ42 in CSF (F (2,18) =45.984, *P*＜0.001), (n= 5 for WT group, n=7 for CAA-Sham group and n=9 for CAA-pd-MCAO group). Scale bar = 200μm/20 μm(zoom). * *P* <0.05; ** *P* <0.01; *** *P* <0.001. ns: non-significance. The data are shown as the mean ± SEM. After performing the Shapiro-Wilk normality test to examine the normal distribution, One-way analysis of variance (ANOVA) was employed to analyze the data pertaining to multiple groups, subsequently, multiple comparisons were conducted using the uncorrected Fisher's LSD test.

LYVE-1 and Aβ staining of the deep cervical lymph nodes (dCLNs) was performed to evaluate lymphatic drainage function. Immunofluorescence results revealed a marked accumulation of Aβ (red) in the dCLNs of CAA mice (16.3%±1.50 vs. 5.8%±0.98, *p*＜0.001, n=7 and 5), which was reduced in the stroke group, as compared to sham CAA controls (7.1%±0.81 vs. 16.3%±1.50, *p*＜0.001, n=9 and 7, Fig. 5A & B, Fig. 5B quantificatio**n**). In parallel, LYVE1+ lymphatic vessel area within the dCLNs was significantly reduced in CAA mice compared to WT (11.6%±1.55 vs. 25.4%±2.72, *p*＜0.001, n=7 and 5), indicating lymphatic structural abnormalities, and this was further reduced following stroke (5.8%±0.96 vs. 11.6%±1.55, *p*=0.013, n=9 and 7, Fig. 5 A & C), suggesting compromised lymphatic function. Consistently, ELISA analysis of cerebrospinal fluid (CSF) demonstrated a significant accumulation of Aβ42 in the CAA group (929.4±55.99 vs. 220.3±6.59, *p*＜0.001, n=7 and 5). Levels were reduced in the stroke group compared to sham CAA and WT mice (724.1±47.5 vs. 929.4±55.99, *p*=0.005, n=9 and 7, Fig. 5D), indicating impaired clearance of brain derived Aβ after stroke.

## DISCUSSION

Our study revealed several novel and important findings. First, we demonstrated that cognitive function, particularly memory performance, is significantly impaired in CAA mice following stroke. Second, we observed a pronounced shift in astrocyte phenotypes within the brainstem RTN region, with increased A1-like reactive astrocytes contributing to aggravated respiratory dysfunction and apnea episodes in the CAA model following stroke. Third, we found that respiratory impairment is associated with compromised lymphatic drainage, as evidenced by reduced LYVE1⁺ lymphatic structures in the dCLNs, reduced Aβ clearance in CSF, and increased Aβ accumulation in the cortex and hippocampus.

The efficient clearance of Aβ peptides from the brain is essential for maintaining CNS homeostasis and preventing neurodegenerative processes such as those observed in CAA and Alzheimer’s disease (AD) [37, 38]. Traditionally, it was believed that the waste products, including Aβ, were primarily cleared via cellular degradation pathways such as phagocytosis and autophagy, or by diffusion into the venous circulation [39]. However, recent clinical trials and basic research have reshaped this paradigm through the identification of the glymphatic system and meningeal lymphatic vessels [40, 41]. The lymphatic system, a brain-wide perivascular fluid transport mechanism, facilitates the convective influx of CSF along periarterial spaces into the brain parenchyma, where it exchanges with interstitial fluid (ISF) and solutes such as Aβ [42]. This interstitial fluid, carrying metabolic wastes, then flows along perivenous spaces back toward the subarachnoid space (SAS), ultimately connecting with the lymphatic network and the systemic circulation [43, 44]. This network provides a physical route for drainage of CSF and Aβ into the deep cervical lymph nodes, thereby coupling the CNS to peripheral immune surveillance and clearance pathways.

Impairment of glymphatic flow has been shown to result in the accumulation of interstitial Aβ, contributing to plaque formation and the progression of amyloid-related pathology [29]. In addition, breathing dysfunction, including apneas, can compromise glymphatic efficiency, highlighting its vulnerability and importance in maintaining brain health [45]. Previous studies in our lab indicated that aged CAA mice exhibit breathing dysfunction and cognitive impairment, which are associated with the astrocytic gliosis in the RTN nucleus [11]. Here, we tested if an ischemic event in CAA mice can further impair RTN-regulated respiratory function, which in turn disrupts glymphatic clearance and exacerbates Aβ deposition.

Firstly, our results showed that ischemic stroke in CAA mice produced exacerbated breathing dysfunction and cognitive impairment. As we previously demonstrated in stroke models, ischemic events can lead to respiratory dysfunction and intermittent hypoxia in mice [19, 24]. Intermittent hypoxia may increase brain amyloid accumulation through multiple mechanisms. For instance, respiratory disturbances can disrupt sleep, thereby impairing glymphatic clearance in the brain and CSF. Chronic intermittent hypoxia may also enhance amyloid production, thereby leading to further impairment of cognitive and memory functions in CAA mice [46, 47], which we will study in the future.

The distal MCAO stroke model produces cortical infarcts without directly injuring brainstem neurons [24]. However, it triggers astrocytic activation in remote brainstem regions, such as the RTN. Our previous work suggested that this is mediated by enhanced TGF-β signaling [27], where microglia-derived TGF-β diffuses globally, activating Smad2/Smad3 pathways in astrocytes and driving chondroitin sulfate proteoglycan (CSPG) overexpression, a key component of glial scarring [48].

Following ischemic stroke, astrocytes polarize into distinct phenotypes [49]. The A1 phenotype, characterized by C3 expression, promotes neurotoxicity through the release of pro-inflammatory factors. In contrast, the A2 phenotype, marked by S100A10 expression, contributes to neuroprotection by producing anti-inflammatory molecules [50, 51]. While previous studies have shown ischemia can induce A2 astrocyte activation in cortical regions [52, 53], our findings in the RTN reveal a paradoxical reduction in S100A10-positive cells alongside an increase in C3 expression. This discrepancy may be due to differences in the brain regions studied, the severity of the infarct, and the timing of assessment.

Both A1 and A2 astrocytes contribute to CSPG production and glial scar formation. However, experimental evidence suggests that A1 astrocytes play a dominant role. For instance, inhibiting the A1 phenotype with calscsoin treatment in LPS-stimulated astrocyte cultures reduced glial scar formation significantly [54]. This highlights the potential therapeutic benefit of targeting A1-specific pathways.

Elevated TGF-β levels correlate with increased C3 expression in dementia patients [55], suggesting a TGF- β may be a contributor to A1 activation. Modulating astrocytic polarization offers a promising therapeutic avenue for ischemic stroke. In the present study, astrocytic phenotypes were evaluated only at a single late time point (42 days post-stroke), which limits our understanding of the temporal evolution of A1/A2 polarization. Previous studies have shown that astrocyte activation after ischemic injury is dynamic and stage-dependent, potentially influencing functional recovery at different phases. Further research should focus on the temporal dynamics of A1/A2 polarization in the RTN and its impact on respiratory function across multiple post-stroke time points (e.g., 7, 14, 28, and 42 days) to better elucidate the temporal profile of reactive astrocytes in the RTN and their contribution to respiratory and cognitive outcomes.

We traced the distribution of Aβ using immunofluorescence and ELISA. In CAA mice, like in patients with CAA, an early-onset and robust age-dependent accumulation of vascular thioflavin-S positive fibrillar Aβ was observed in the thalamus, inferior colliculus, and hippocampus, as seen in both early- and late-stage sporadic vascular dementia, while widespread diffuse parenchymal Aβ deposition was detected in the cortex [56-58]. Consistent with the distribution pattern of Aβ observed in CAA mice and CAA patients, our immunofluorescence results further confirmed that stroke significantly aggravates Aβ deposition in both the hippocampus and cortex. These results indicate that ischemic stroke exacerbates brain amyloid pathology in key brain regions associated with cognition and memory.

As discussed above, this accumulation of Aβ may be attributed to impaired clearance mechanisms induced by breathing dysfunction, including respiratory frequency and apneas, both of which are closely linked to perivascular drainage. The regional specificity of amyloid aggregation, particularly in the hippocampus and cortex, underscores the vulnerability of these areas to combining vascular and ischemic insults, potentially accelerating cognitive decline in comorbid CAA and stroke conditions.

Interestingly, while we observed a significant increase in Aβ deposition within the hippocampus and cortex following stroke, the levels of Aβ detected in the CSF and dCLVs were significantly reduced. This inverse pattern suggests that stroke impairs the glymphatic clearance capacity in CAA mice. Under normal conditions, the glymphatic system facilitates the transport of interstitial Aβ from the brain parenchyma into the CSF and ultimately drains it into peripheral lymphatic structures, such as the deep cervical lymph nodes [59, 60]. Such impairment may result from stroke-induced disruptions to respiratory-driven CSF dynamics, which are critical for glymphatic circulation. Consequently, inefficient Aβ clearance exacerbates its accumulation in key cognitive regions, contributing to the progression of amyloid pathology and cognitive decline. These findings underscore the essential role of glymphatic-lymphatic drainage in maintaining brain homeostasis and highlight its vulnerability in the context of CAA compounded by stroke.

Although our study suggests an association between RTN astrogliosis and impaired glymphatic clearance, the mechanistic link remains indirect. RTN dysfunction may contribute to altered respiratory rhythms, such as increased apnea frequency, hypoventilation, and instability [61], which in turn could impact systemic venous return and generate irregular intracranial pressure (ICP) fluctuations [62-64]. Since CSF flow within the glymphatic system is partially driven by arterial pulsatility and pressure dynamics, disrupted respiratory-driven ICP modulation may impair CSF circulation and thereby reduce glymphatic clearance efficiency. Future studies measuring ICP changes in relation to RTN activity could further elucidate this pathway.

In this study, there are several limitations that warrant further investigation in future research. Firstly, this experiment was conducted exclusively using female mice, which may limit the generalizability of the findings. Sex hormones can influence cerebrovascular function [65], breathing function [66], neuroinflammation [67], and glymphatic clearance efficiency [68], therefore it is not scientifically rigorous to intermix females and males in the same cohorts for this study. Research indicates that female CAA mice exhibit worse cognitive impairment and more severe pathology compared to male mice [69]. Similarly, in human patients, female individuals with CAA or Alzheimer's disease often experience more severe cognitive impairment and pathology. Therefore, we chose to start with female mice first. Future studies should include both male and female animals to fully elucidate sex-dependent differences in CAA progression and stroke-induced pathology.

Second, although we identified changes in astrocytic A1/A2 phenotypes in the RTN region, the temporal evolution of these phenotypic transitions following stroke was not systematically tracked. A more detailed time-course analysis (e.g., 7, 14, 28, 42 days post-stroke) would help clarify the dynamic role of astrocytes in regulating respiratory dysfunction and Aβ accumulation. Third, the study inferred glymphatic impairment was inferred indirectly based on decreased Aβ levels in the CSF and dCLNs, along with increased parenchymal deposition. However, direct measurements of glymphatic flow were not performed, representing a limitation of the current study. Techniques such as intrathecal tracer injection, two-photon microscopy, or *in vivo* imaging (e.g., MRI with Gd tracers) could provide more definitive evidence of glymphatic dysfunction in future studies. incorporating these techniques would strengthen the mechanistic interpretation of how respiratory dysfunction and astrocytic reactivity impair glymphatic clearance.

In conclusion, our study reveals a novel link between stroke-induced impairment of glymphatic clearance and aggravated Aβ accumulation in CAA mice. We demonstrate that respiratory dysfunction, particularly involving astrocytic reactivity and transformation in the RTN, plays a critical role in disrupting brain waste clearance pathways. These findings highlight the importance of maintaining glymphatic function in cerebrovascular diseases and suggest that targeting astrocyte-mediated mechanisms may offer a promising therapeutic approach.

## Data Availability

The datasets and materials used and/or analyzed during the current study are available from the corresponding author on reasonable request.
